# s-ICAM-1 and s-VCAM-1 in healthy men are strongly associated with traits of the metabolic syndrome, becoming evident in the postprandial response to a lipid-rich meal

**DOI:** 10.1186/1476-511X-7-32

**Published:** 2008-09-01

**Authors:** Diana Rubin, Sandra Claas, Maria Pfeuffer, Michael Nothnagel, Ulrich R Foelsch, Juergen Schrezenmeir

**Affiliations:** 1 Max-Rubner-Institut, Federal Research Center of Nutrition and Food, Herrmann-Weigmann-Str.1, 24103 Kiel, Germany; 2 Department of Surgery, Knappschaftskrankenhaus Bochum-Langendreer, Ruhr Universität Bochum, Germany; 3 Institute of Medical Informatics and Statistics, University of Kiel, Brunswiker Str. 10, 24105 Kiel, Germany; 4 Department of General Internal Medicine, University Clinic Schleswig-Holstein, Campus Kiel, Schittenhelmstr. 12, 24105 Kiel, Germany

## Abstract

**Background:**

The importance of the postprandial state for the early stages of atherogenesis is increasingly acknowledged. We conducted assessment of association between postprandial triglycerides, insulin and glucose after ingestion of a standardized lipid-rich test meal, and soluble cellular adhesion molecules (sCAM) in young healthy subjects.

**Methods:**

Metabolic parameters and sICAM-1, sVCAM-1 and E-selectin were measured before and hourly until 6 hours after ingestion of a lipid-rich meal in 30 healthy young men with fasting triglycerides <150 mg/dl and normal fasting glucose levels. Subjects were classified as either normal responders (NR) (postprandial triglyceride maxima < 260 mg/dl) or high responders (HR) (postprandial triglyceride maxima > 260 mg/dl). Levels of CAM were compared in HR and NR, and correlation with postprandial triglyceride, insulin and glucose response was assessed.

**Results:**

Fasting sICAM-1 and sVCAM-1 levels were significantly higher in HR as compared to NR (p = 0.046, p = 0.03). For sE-selectin there was such a trend (p = 0.05). There was a strong positive and independent correlation between sICAM-1 and postprandial insulin maxima (r = 0.70, p < 0.001). sVCAM-1 showed significant correlation with postprandial triglycerides (AUC) (r = 0.37, p = 0.047). We found no correlation between sCAMs and fasting insulin or triglyceride concentrations.

**Conclusion:**

This independent association of postprandial triglycerides with sICAM-1 may indicate a particular impact of postprandial lipid metabolism on endothelial reaction.

## Background

Coronary artery disease is the main cause of death in middle-aged men [1] and atherosclerosis is responsible for 50% of all mortality in the USA, Europe and Japan [2]. There are several theories about the pathogenesis of atherosclerosis but the ‚response-to-injury‘-hypothesis of R. Ross [2,3] is widely accepted. Several different sources of injury to the endothelium lead to endothelial cell dysfunction. The initial stages of atherosclerosis are characterized by adhesion of circulating leukocytes to the endothelial cells and subsequent transendothelial migration. This process is mediated in part by cellular adhesion molecules (CAMs) like vascular cell adhesion molecule-1 (VCAM-1), intercellular adhesion molecule-1 (ICAM-1), and E-selectin, expressed on the endothelial membrane, in response to several inflammatory cytokines, including interleukin-1, tumor necrosis factor and interferon [4]. Cellular adhesion molecules are later on detached from the membrane and are found in the circulation in their soluble form. CAM expression is increased in atherosclerotic plaques [5,6]. Soluble forms of these adhesion molecules (sVCAM-1, sICAM-1, sE-selectin) can be detected in the serum and are increased in conditions with an inflammatory component [7,8] and in atherosclerosis resulting in coronary artery disease [9-11], carotid sclerosis [10] and peripheral vascular disease [12]. Furthermore increased levels of sCAMs are associated with components of the metabolic syndrome like diabetes mellitus [13], hypertension [14] and dislipidemia [15,16]. A slight but significant independent correlation of sCAMs and fasting triglycerides has been observed [15,17]. The impact of postprandial metabolism on sCAMs has been shown during an oral glucose tolerance test, when postprandial insulin levels correlated directly with sICAM-1 [18]. Another study compared 2 h and 4 h postprandial levels of sICAM-1 and sVCAM-1 after a high-fat versus a high-carbohydrate diet in diabetic and normal subjects [19]. In this study, an increase of ICAM-1 and VCAM-1 occurred after high-fat meal in non-diabetic subjects and was prevented by addition of vitamine E and C. Two other studies showed that soluble adhesion molecules were increased after a high fat meal [20,21]. There are no previous reports on the relation between postprandial triglycerides, glucose, insulin and soluble adhesion molecules after a standardized lipid load in healthy subjects.

The aim of the study was to evaluate soluble adhesion molecules sICAM-1, sVCAM-1 and sE-selectin after a mixed meal in young healthy subjects with normal or high triglyceride response to the meal.

## Results

The characteristics of the study population are shown in Table 1. High responders showed significantly higher fasting triglycerides and insulin concentrations (p < 0.01, p = 0.039, rsp.), as well as higher fasting sICAM-1, sVCAM-1 and sELAM-1 (p = 0.046, p = 0.034, p = 0.05, rsp.) concentrations.

**Table 1:** Characteristics of the study population

	Normal Responder (n = 15)	High Responder (n = 15)	P-value
Age (years)	24.27 ± 2.84	25.73 ± 2.79	0.23
BMI (kg/m^2^)	22.43 ± 1.82	24.15 ± 2.86	0.12
WHR	0.81 ± 0.05	0.85 ± 0.06	0.08
Fasting triglycerides (mg/dl)	79.4 26.1	129.1 ± 31.3	<0.001
Fasting glucose (mg/dl)	94.1 ± 16.5	88.3 ± 9.9	0.26
Fasting insulin (mU/l)	15.3 ± 6.5	10.9 ± 4.4	0.039
Fasting sICAM-1 (ng/ml)	217.2 ± 40.8	307.3 ± 79.3	0.046
Fasting sVCAM-1 (ng/ml)	320.6 ± 177.4	418.7 ± 121.0	0.034
Fasting sELAM-1 (ng/ml)	13.2 ± 7.4	18.4 ± 9.6	0.050

Anthropometric variables, fasting metabolic parameters, and soluble adhesion molecules in 15 normal and 15 high triglyceride responders (mean ± SD).

### Glucose

**Figure 1:**
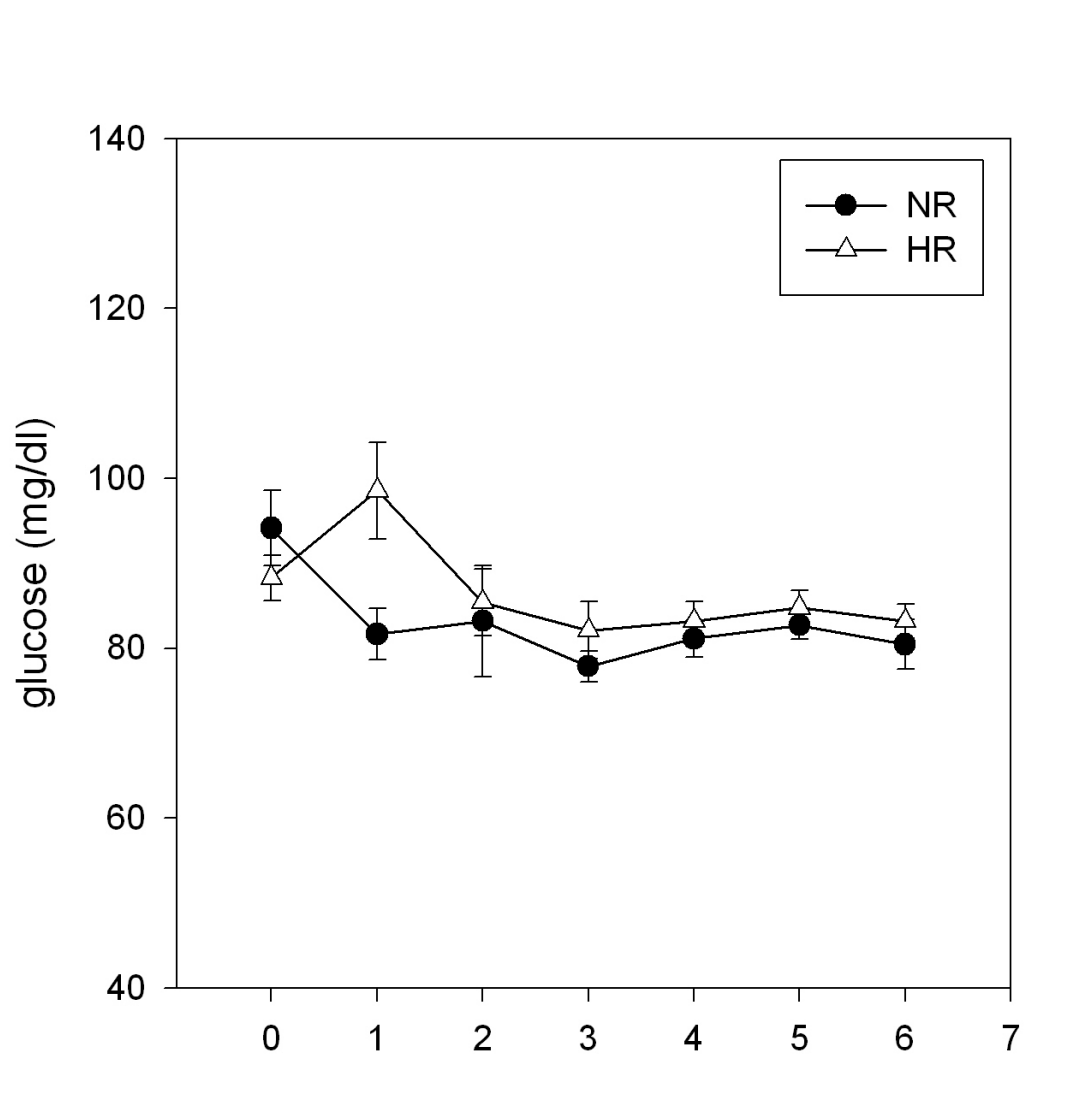
**Postprandial concentrations of glucose**. Plasma glucose concentrations following ingestion of an oral metabolic tolerance test in 15 normal (●, NR) and 15 high (△, HR) triglyceride responders (mean ± SEM).

None of the subjects showed impaired glucose tolerance. Fasting and postprandial (AUC) glucose levels were not different between groups. In NR, the glucose concentration falls below fasting 60 min after the oMTT, in HR an increase of glucose after 60 min resulted in a significant difference at this time point (p = 0.01) (Figure 1).

### Insulin

**Figure 2:**
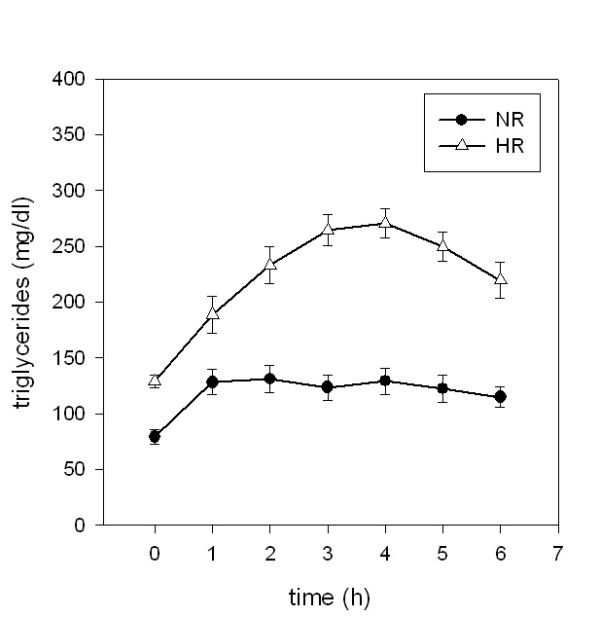
**Postprandial concentrations of insulin**. Plasma insulin concentrations following ingestion of an oral metabolic tolerance test in 15 normal (●, NR) and 15 high (△, HR) triglyceride responders (mean ± SEM).

Fasting insulin levels were significantly different in normal and high responders (p = 0.04) with higher concentrations for normal responders. After ingestion of the test meal, there was a tendency towards higher insulin levels in HR (p = 0.10 for AUC) (Figure 2).

### Triglycerides

**Figure 3:**
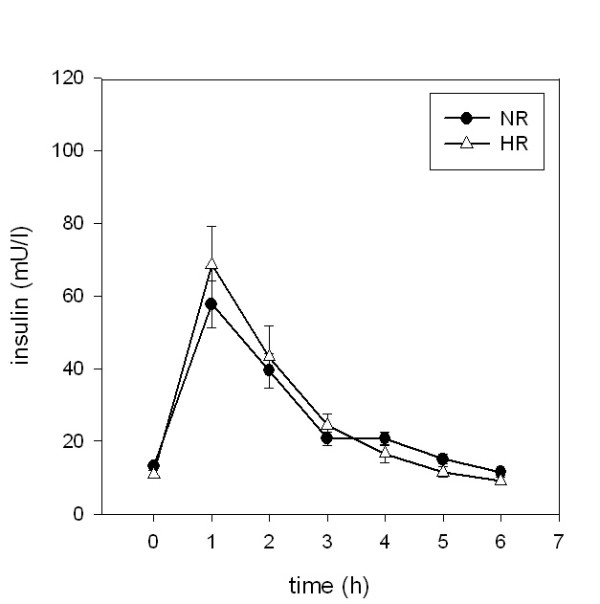
**Postprandial concentrations of triglycerides**. Plasma triglyceride concentrations following ingestion of an oral metabolic tolerance test in 15 normal (●, NR) and 15 high (△, HR) triglyceride responders (mean ± SEM).

Fasting and following the ingestion of the oral metabolic tolerance test, mean plasma triglyceride levels were significantly higher in high responders as compared to normal responders at any time point (p < 0.001) (Figure 3).

### Soluble adhesion molecules

**Figure 4:**
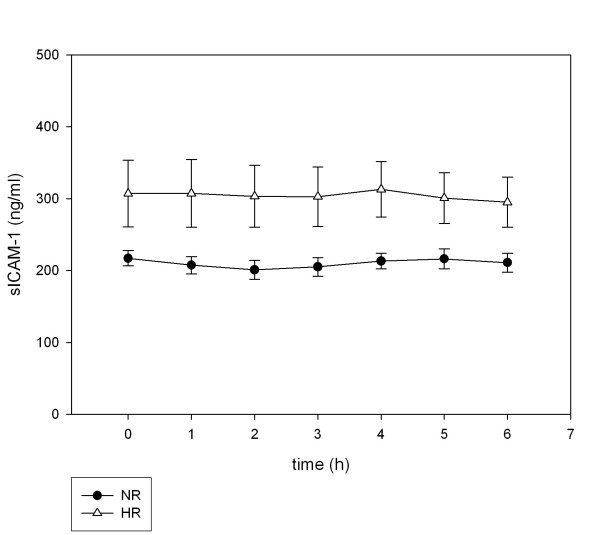
**Postprandial concentrations of sICAM-1**. Plasma sICAM-1 concentrations following ingestion of an oral metabolic tolerance test in 15 normal (●, NR) and 15 high (△, HR) triglyceride responders (mean ± SEM).

**Figure 5:**
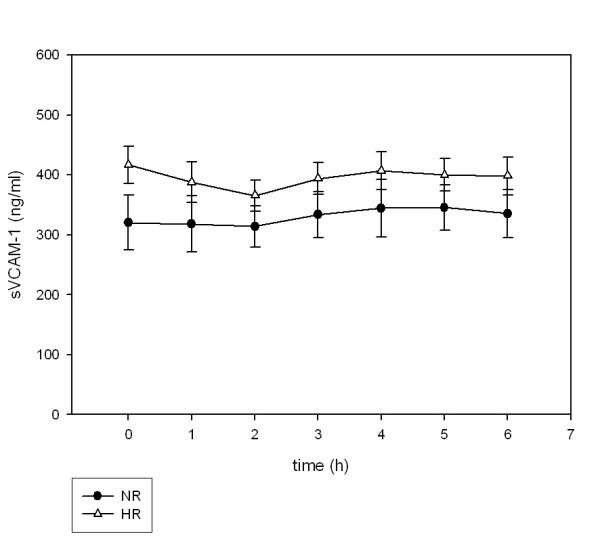
**Postprandial concentrations of sVCAM-1**. Plasma sVCAM-1 concentrations following ingestion of an oral metabolic tolerance test in 15 normal (●, NR) and 15 high (△, HR) triglyceride responders (mean ± SEM).

**Figure 6:**
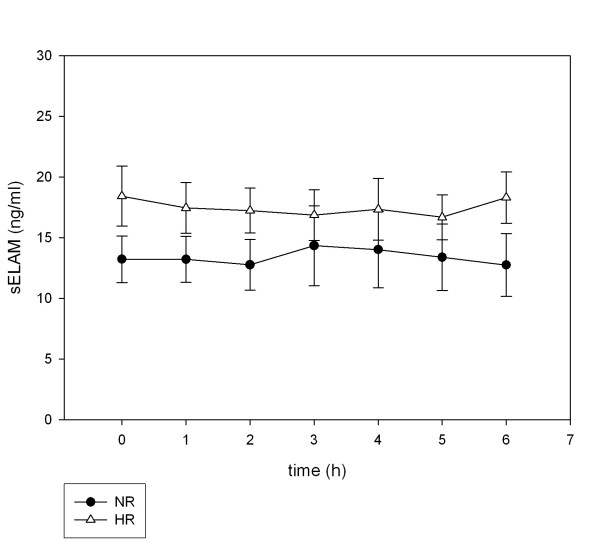
**Postprandial concentrations of E-Selectin**. Plasma E-Selectin concentrations following ingestion of an oral metabolic tolerance test in 15 normal (●, NR) and 15 high (△, HR) triglyceride responders (mean ± SEM).

Soluble adhesion molecules (sICAM-1, sVCAM-1, sE-selectin) did not increase in the postprandial state (Figure 4, 5, 6). Neither the levels at single postprandial time points nor the means over observation time differed from fasting concentrations for sICAM-1, sVCAM-1 and sE-selectin. Therefore, the fasting values were used to analyze differences between NR and HR. sICAM-1 levels were significantly different between NR (210.2 ± 5.94 ng/ml) and HR (304.3 ± 5.69 ng/ml, p = 0.046). Plasma levels of sVCAM-1 were also significantly higher in HR (p = 0.047). Soluble E-selectin levels were not significantly different but tended to be higher in HR (18.4 ± 9.6 vs. 13.2 ± 7.4, p = 0.05).

### Relationship of sCAM and metabolic parameters

In univariate linear regression analysis, fasting soluble ICAM-1 correlated with with postprandial insulin AUC and the maximum of insulin after oMTT (r = 0.39, p < 0.04, r = 0.7, p < 0.001 rsp.). There was no significant correlation with fasting insulin concentration. Correlation between fasting sVCAM-1 and postprandial triglycerides again showed a stronger correlation (r = 0.37) than with fasting triglycerides (r = 0.34, p = 0.047). There was no significant correlation between fasting sE-selectin and fasting or postprandial glucose, insulin or triglycerides (Table 2).

**Table 2:** Correlation coefficients of soluble adhesion molecules with fasting and postprandial metabolic parameters

	sICAM-1	sVCAM-1	sELAM-1
Fasting glucose	-0.070	0.042	-0.088
Glucose max	0.073	0.147	0.241
Glucose AUC	0.089	0.189	0.057
Fasting insulin	-0.038	0.047	-0.185
Insulin max	0.699*	0.013	0.071
Insulin AUC	0.385*	0.249	-0.004
Fasting triglycerides	0.212	0.337	0.087
Triglycerides max	0.290	0.316	0.310
Triglycerides AUC	0.288	0.366*	0.164

Correlation coefficients of fasting and postprandial glucose, insulin, and triglycerides assessed by univariate linear regression analysis following ingestion of an oral metabolic tolerance test in 30 healthy men. The numeric values are standardized regression coefficients. *significant correlation in univariate analysis

In multivariate linear regression analysis, including fasting, maximal and AUC-values of insulin, glucose, and triglycerides, we found a strong prediction of fasting sICAM-1 through triglycerides (AUC), insulin maxima, and postprandial glucose (AUC) (r^2 ^0.67, p < 0.001). 67% of the variance of fasting sICAM-1 concentration was predicted by these three parameters, although the latter contributed to the model goodness but was not significant as an independent predictor for fasting sICAM-1. For fasting sE-selectin and sVCAM-1, the metabolic parameters were much weaker predictors. Only 8% at most of the variance of fasting sE-selectin and sVCAM-1 where predicted when metabolic parameters were included. sE-selectin was predicted significantly through postprandial triglycerides which remained in the model with a borderline significance for maximal triglycerides (p = 0.08), while for sVCAM-1 none of the included metabolic parameter was an significant predictor.

## Discussion

In this study we showed, that healthy young men with normal fasting triglycerides but high postprandial triglycerides (HR) showed higher levels of sCAMs after a lipid-rich meal compared to subjects with lower postprandial triglycerides (NR). Furthermore, HR are characterized by higher postprandial glucose concentrations, as important early characteristics of the metabolic syndrome, whereas fasting glucose was low in these subjects. The NR showed an initial decrease of glucose concentrations after oMTT which is a sign of intact glucose-insulin regulation and was significantly different from the 60 min increase of glucose in HR.

Ferri et al. [18] found an increase of sICAM-1 after an oral glucose tolerance test and a correlation with postprandial insulin in subjects with impaired glucose tolerance and hyperlipidemia. Furthermore subjects with isolated hyperlipidemia had normal levels of sICAM in their study. The authors concluded that glucose metabolism and insulin resistance, not hyperlipidemia, has direct impact on levels of sCAM. We showed that in fact postprandial insulin correlates with sICAM. The strongest correlation was seen between both insulin maxima and the insulin area-under-the-curve and sCAMs, especially for sICAM-1. But as there was no postprandial augmentation of sCAM in our study, the conclusion of Ferri et al. that insulin has direct impact on CAMs is not supported by our results. Instead we conclude that there is a general metabolic abnormality seen in high responders which may be an abnormality which eventually may lead to the inflammatory abnormalities accompanying the metabolic syndrome.

High responders showed higher postprandial lipid peroxidation and reduced intracellular levels of antioxidant vitamin C in previous studies [22]. The association between high postprandial triglyceride and insulin levels after a mixed meal with high CAMs is presumably indicating an early stage of endothelial dysfunction.

In this study strong correlations between postprandial insulin levels and sICAM were seen whereas the correlation between fasting insulin levels and sICAM was less pronounced. Moreover, the linear relationship between sICAM, postprandial triglycerides, and insulin maxima was independent of fasting metabolic parameters in multiple regression analysis. Again, postprandial triglyceride levels showed a significant correlation to sVCAM, while this correlation was not found with fasting triglycerides. In contrast, Ridker et al. [9] found a slight but significant correlation between sICAM-1 and fasting triglycerides in patients with risk for future myocardial infarction. In this study, the postprandial metabolism was not examined. The important role of the postprandial state for induction of early stages of metabolic syndrome and atherosclerosis is evident because of stronger and independent postprandial correlations.

Intervention studies showed that metabolic disorder as well as diet influence CAM levels and, thus, the endothelial activation. In poorly controlled NIDDM diabetics, increased sE-selectin levels returned to normal after short-term improvement of glycemic control [23]. In other studies, lipid lowering therapy decreased levels of sE-selectin in hypercholesteremic patients [15] and n3-fatty acid treatment decreased sE-selectin and sICAM-1 [17] in hypertriglyceridemic patients.

According to sICAM-1, we also found higher levels of fasting sVCAM-1 in high responders compared to normal responders. sE-selectin concentrations were also higher in HR, although the difference was statistically not significant. In unstimulated endothelium, adhesion molecule expression is low, with the exception of ICAM-1, which is constitutively expressed to a higher degree. Because of the "premetabolic" syndrome in high responders, we assume that ICAM-1 is expressed constantly on endothelial cells in these persons in a higher degree than E-selectin and VCAM, which can explain the most pronounced difference in HR and NR for sICAM-1.

Correlation analysis of any CAMs with postprandial glucose in our collective was negative, implicating the importance of insulin and triglycerides in early stages of metabolic syndrome when glucose levels are still normal.

In our subjects there was no evidence of clinical manifested atherosclerosis disease, although early stages of atherosclerosis can not be excluded. HR had a tendency for both higher sCAM levels and elevated postprandial triglyceride and insulin levels. We showed for the first time a correlation between postprandial triglycerides, insulin and sCAMs in healthy subjects. Thus, it may be suggested that postprandial triglycerides and/or insulin and sCAMs are reasonable markers for early metabolic abnormalities and endothelial activation leading to the metabolic syndrome and atherosclerosis, however further studies are needed to confirm this. A limitation of this study is the limited sample size which could explain the tendency for elevated sE-selectin in HR, but without reaching statistical significance. Further studies with alterations of oxidative agens in oral lipid loads are needed to examine postprandial levels of adhesion molecules.

In this study soluble adhesion molecules did not increase within the 6 h observation period after an oral lipid load. Other investigators found increased levels of CAM as early as 2 hours after a glucose load [19]. An increase of soluble adhesion molecules after high fat meals was shown in other studies [20,21]. Incubation of HUVEC endothelial cells with chylomicrons can induce E-selectin and VCAM-1 expression [24,25].

We assume that the retinol content of our test meal attenuated the postprandial rise of soluble adhesion molecules. We did indeed observe that consumption of the lipid load without retinol increases postprandial levels of sICAM and sE-selectin, and that this effect is prevented by retinol (Pfeuffer et al., personal communication). This fits with the results of Nappo et al. [19]. When these authors administered a high-fat diet meal with or without supplementation of vitamin E and C as antioxidative agents to diabetic and healthy subjects, the high-fat meal without antioxidants significantly increased postpranadial levels of sICAM-1 and sVCAM-1. This effect was prevented by addition of vitamin E and C in both groups.

## Conclusion

In conclusion, soluble adhesion molecules are strongly associated with postprandial but not fasting triglycerides and insulin in young, healthy men which could have implications for future atherogenic risk assessment in healthy subjects, but further studies are needed to confirm this.

## Methods

### Subjects

Thirty healthy male subjects with normal fasting triglycerides (<150 mg/dl) were recruited according to their postprandial response to a standardized lipid-rich meal. In a previous study, a bimodal distribution of triglyceride maxima following an oral lipid load was observed. In total 15% of the subjects did respond with postprandial triglyceride maxima above 260 mg/dl. The cut-off point to identify the high responders (HR) was chosen according to a bimodal distribution of triglyceride maxima [26,27]. Fifteen normal responders (NR) and fifteen HR were selected out of 182 subjects who underwent the standardized lipid-rich meal in the current study. Inclusion criteria were normal BMI (<25 kg/m^2^) and fasting blood glucose levels < 110 mg/dl. Subjects had no history of present or past hypertension, hyperlipidemia, diabetes, or cardiovascular disease. All subjects were following previously ad libidum diets, had no recent change in body weight or intercurrent illness and were taking no medication. The study complies with the Declaration of Helsinki. The protocol of the study was approved by the ethics commitee of the University of Kiel, the subjects gave informed written consent before being tested. The characteristics of the study population are reported in table 1.

### Standardized lipid-rich meal

Studies began at 8 AM after a 12-h-overnight fast. After fasting blood was drawn, the subjects consumed 500 ml of a standardized mixed liquid meal (oral metabolic tolerance test, oMTT) containining the following ingredients: 30 g of protein (11,9 energy%), 75 g of carbohydrates (29,6 energy%)(93% saccharose, 7% lactose), 58 g of fat (51,6 energy%)(65% saturated, 35% unsaturated fatty acids), 10 g of alcohol (6,9 energy%), 600 mg cholesterol and 30.000 IU retinylpalmitate (Nutrichem, Roth, Germany). The total energy content was 1017 kcal (4255 kJ). The test meal was drunk within 15 minutes. Blood withdrawal was repeated at 1, 2, 3, 4, 5 and 6 h after ingestion of oMTT. Subjects were allowed to walk or sit, as they wished, but not to exercise during the test. Ad libitum drinking of water or fruit tea without sugar was permitted. Blood was collected through a venous in-dwelling catheter placed in a cubital vein. For assessing glucose tubes containing 1 mg/ml fluoride and 1.2 mg/ml EDTA, for determing triglycerides, insulin and cellular adhesion molecules tubes containing 1.6 mg/ml Potassium EDTA were used. Plasma was separated by centrifugation at 6.48 e+7/min, 4°C and stored at -20°C until analysis.

### Laboratory analyses

Fasting and postprandial triglyceride and glucose were determined automatically and in duplicate with the kone lab 20i analyzer (Kone, Espoo, Finland) using enzymatic test kits (glucose: Roche, Mannheim, Germany; triglycerides: Boehringer, Mannheim, Germany). Insulin was measured with a radioimmunassay (Biochem Immunosystems, Freiburg, Germany). Plasma concentrations of sICAM-1, sVCAM-1 and sE-selectin were measured in duplicate using a quantitative sandwich ELISA (Boehringer, Mannheim, Germany). The inter-assay and intra-assay coefficients of variation were < 10%.

### Statistical analyses

The Kolmogorov-Smirnov test was used to determine whether each considered variable had a normal distribution. Comparisons of baseline data among the groups were performed using the unpaired Student's *t *test for normally distributed parameters and with the Mann-Whitney-U test for parameters not following a normal distribution. The paired Student's *t *test was used for comparison of CAMs before and after ingestion of the test meal. If differences reached statistical significance, post-hoc analysis with a two-sided paired *t *test was used to assess differences at individual time periods in the study, using Bonferroni correction to adjust for for multiple comparisons. The statistical significance of postprandial change of sCAMs was determined by comparing the summarized postprandial values (area under the curve, AUC) with the fasting values by a t test or a Mann-Whitney-U test, depending on the presence of a normal distribution. Spearman's coefficient was used to describe correlations of pooled data from NR and HR. In further exploratory analyses, multiple linear regression analysis was used to examine the relative contributions and overlap of metabolic factors possibly contributing to sCAM levels. Results were given as mean ± SEM. The 0–9 h AUC was calculated by the trapezoidal method [28]. Statistical significance was defined as p < 0.05.

## Competing interests

The authors declare that they have no competing interests.

## Authors' contributions

DR, MP, and JS were responsible for the study design; DR, and SR were responsible for data collection; DR, and MN were responsible for data analysis; and DR, MP, MN, and JS were responsible for writing the manuscript. All authors read and approved the final manuscript.
